# Prevalence of Amyotrophic Lateral Sclerosis — United States, 2015

**DOI:** 10.15585/mmwr.mm6746a1

**Published:** 2018-11-23

**Authors:** Paul Mehta, Wendy Kaye, Jaime Raymond, Reshma Punjani, Theodore Larson, Jessica Cohen, Oleg Muravov, Kevin Horton

**Affiliations:** 1 Division of Toxicology and Human Health Sciences, Agency for Toxic Substances and Disease Registry, CDC.

Amyotrophic lateral sclerosis (ALS), commonly known as Lou Gehrig’s disease, is a progressive and fatal neuromuscular disease; the majority of ALS patients die within 2–5 years of receiving a diagnosis (*1*). Familial ALS, a hereditary form of the disease, accounts for 5%–10% of cases, whereas the remaining cases have no clearly defined etiology (*1*). ALS affects persons of all races and ethnicities; however, whites, males, non-Hispanics, persons aged ≥60 years, and those with a family history of ALS are more likely to develop the disease (*2*). No cure for ALS has yet been identified, and the lack of proven and effective therapeutic interventions is an ongoing challenge. Treatments currently available, Edaravone and Riluzole, do not cure ALS, but slow disease progression in certain patients (*3*,*4*). This report presents National ALS Registry findings regarding ALS prevalence in the United States for the period January 1–December 31, 2015. In 2015, the estimated prevalence of ALS cases was 5.2 per 100,000 population with a total of 16,583 cases identified. Overall, these findings are similar to the 2014 ALS prevalence and case count (5.0 per 100,000; 15,927 cases) (*2*). Prevalence rates by patient characteristics (most common in whites, males, and persons aged ≥60 years) and U.S. Census regions are consistent with ALS demographics and have not changed from 2014 to 2015 calendar years. The algorithm used to identify cases from national administrative databases was updated from the *International Classification of Diseases, Ninth Revision* (ICD-9) to the ICD-10 codes for claims starting on October 1, 2015, with no apparent effect on case ascertainment. Data collected by the National ALS Registry are being used to better describe the epidemiology of ALS in the United States and to facilitate research on the genetics, potential biomarkers, environmental pollutants, and etiology for ALS.

In 2008, the U.S. Congress passed the ALS Registry Act, which authorized the creation and maintenance of the National ALS Registry (Registry), and data collection began in 2010.* The Registry’s goals and methods were described in detail previously (*5*). Because ALS, like most noncommunicable diseases, is not a nationally notifiable condition, cases in the United States are identified using a novel two-pronged approach. The first approach identifies cases from three large national administrative databases (Medicare, Veterans Health Administration, and Veterans Benefits Administration) by using an algorithm with elements such as the ICD code for ALS, frequency of visits to a neurologist, and prescription drug use. On October 1, 2015, ICD-10 codes were integrated into the algorithm, which categorizes cases in Registry nomenclature as “definite ALS,” “possible ALS,” and “not ALS” (*6*). Only definite ALS cases are entered into the Registry. The second approach is a secure web portal that enables persons with ALS to enroll in the Registry, thereby identifying cases not recorded in the national databases. The web portal also allows enrollees the opportunity to complete up to 17 different brief risk-factor modules to describe their experience (e.g., occupational and military histories, smoking and alcohol use, family history of neurologic conditions, and head and neck injuries). Cases from both sources are then merged and deduplicated. Once an ALS case is identified, the patient remains a case until confirmed deceased through the National Death Index. This is referred to as cumulative prevalence of ALS and is calculated from the Registry by using the deduplicated total number of persons with ALS identified through the two-pronged approach for the numerator. The 2015 U.S. Census estimate is used for the denominator and 95% confidence intervals are calculated (*7*).

In 2015, a total of 16,583 persons were identified as having definite ALS by applying the algorithm to the three national databases (62% of ALS cases), by self-report through the web portal registration (19%), and from information in both database and portal (19%) (Table). Overall, 6,250 new ALS cases were identified in 2015, and 5,594 deaths among persons with ALS whose data were included in the Registry during 2014, for a net increase of 656 cases compared with 2014. No apparent difference in the number of ALS cases ascertained in 2014 and 2015 occurred when either ICD-9 or ICD-10 codes were used in each calendar year.

**TABLE:** Number and percentage of amyotrophic lateral sclerosis (ALS) cases (N = 16,583) and estimated prevalence, by age group, sex, race and geographic region — National ALS Registry, United States, 2015

Characteristic	Population*	No. (%) cases	Estimated cases per 100,000 population (95% CI)
**Age group (yrs)**			
18–39	95,782,809	480 (2.9)	0.5 (0.5–0.6)
40–49	41,141,609	1,462 (8.8)	3.6 (3.4–4.1)
50–59	43,712,960	3,214 (19.4)	7.4 (6.9–7.9)
60–69	35,356,070	4,774 (28.8)	13.5 (12.9–14.1)
70–79	19,606,548	3,953 (23.8)	20.2 (19.4–21.3)
≥80	11,892,496	1,522 (9.2)	12.8 (12.3–13.4)
Unknown	—	1,178 (7.1)	—
**Sex**			
Male	158,138,060	10,098 (60.9)	6.4 (6.2–6.5)
Female	163,280,761	6,458 (38.9)	4.0 (3.9–4.1)
Unknown	—	27 (0.16)	—
**Race**			
White	243,635,466	13,074 (78.8)	5.4 (5.2–5.6)
Black	44,677,216	1,045 (6.3)	2.3 (2.2–2.5)
Other	—	958 (5.8)	—
Unknown	—	1,503 (9.1)	—
**U.S. Census region^†^**			
Midwest	67,907,403	3744 (25.6)	5.5 (5.4–5.6)
Northeast	56,283,891	2881 (17.4)	5.1 (5.0–5.2)
South	121,182,847	5676 (34.2)	4.7 (4.6–4.8)
West	76,044,679	3352 (20.2)	4.4 (4.3–4.5)
Unknown	—	930 (5.6)	—
**Total**	**321,418,821**	**16,583 (100.0)**	**5.2 (5.1–5.3)**

**Abbreviation:** CI = confidence interval.

* From 2015 U.S. Census data.

^†^ *Northeast:* Connecticut, Maine, Massachusetts, New Hampshire, New Jersey, New York, Pennsylvania, Rhode Island, Vermont; *South*: Alabama, Arkansas, Delaware, District of Columbia, Florida, Georgia, Kentucky, Louisiana, Maryland, Mississippi, North Carolina, Oklahoma, South Carolina, Tennessee, Texas, Virginia, West Virginia; *Midwest*: Iowa, Illinois, Indiana, Kansas, Michigan, Minnesota, Missouri, Nebraska, North Dakota, Ohio, South Dakota, Wisconsin; *West*: Alaska, Arizona, California, Colorado, Hawaii, Idaho, Montana, Nevada, New Mexico, Oregon, Utah, Washington, Wyoming.

The 2015 estimated prevalence of ALS cases was 5.2 per 100,000 population, which is similar to the 2014 prevalence (5.0). The prevalence across age groups appears to be stable (Figure). The lowest prevalence (0.5 ALS cases per 100,000 population) was among persons aged 18–39 years, and the highest (20.2) was among persons aged 70–79 years (Table). As in 2014, the prevalence in males (6.4 ALS cases per 100,000 population) was higher than that in females (4.0). The ratio of cases in males to females was 1.6:1. The prevalence in whites (5.4 ALS cases per 100,000 population) was more than twice that in blacks (2.3). Prevalence rates were also calculated for the four U.S. Census regions (Northeast, South, Midwest, and West). Rates were highest in the Midwest (5.5 ALS cases per 100,000 population), followed by the Northeast (5.1), the South (4.7), and the West (4.4).

**FIGURE:**
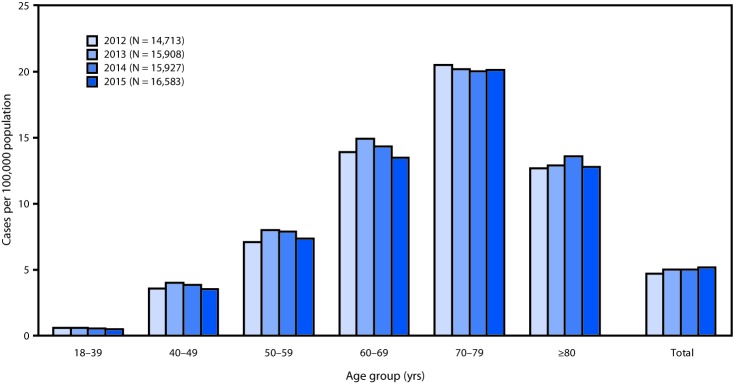
Estimated prevalence of amyotrophic lateral sclerosis (ALS), by age group — National ALS Registry, United States, 2012–2015* * Prevalence per 100,000 population using the 2015 U.S. Census estimate.

## Discussion

Data sources for the Registry remain unchanged; however, the implementation of ICD-10 on October 1, 2015 required that ICD-10 codes be integrated into the validated algorithm without any apparent effect on case ascertainment. The Registry’s approach of using national administrative databases is the cornerstone in identifying ALS cases because most of the definite ALS cases from 2010 to 2015 originate from these sources.

ALS has remained more prevalent in whites, males, and persons aged ≥60 years; current patterns are similar to those identified from 2010 to 2014 (*2*–*4*). The prevalence of ALS cases for 2015 appears to be stable (5.2 per 100,000 compared with 5.0 per 100,000 for 2014). The net increase of 656 cases is likely attributable to additional case ascertainment from the administrative databases, specifically Medicare, because the accumulation of data over multiple years might be adequate to finally meet the algorithm-based ALS case definition. This slight change in prevalence does not necessarily indicate an increase of ALS cases nationally. Additional years of data are needed to evaluate any possible trends. The Registry continues to evaluate additional data sources for case identification as well as ways to increase self-enrollment through the secure web portal to improve case ascertainment.

Prevalence rates by U.S. Census regions are consistent with ALS demographics and have not changed from 2014 to 2015 calendar years. Overall, whites have a higher prevalence of ALS than do blacks. The higher ALS prevalence in the Midwest and Northeast likely reflects the higher proportion of whites in those regions, compared with that in the South and West. The lowest prevalence in the West Census region is most likely related to the population diversity in states such as California.

In addition to monitoring the epidemiology of the disease, the Registry continues to expand and facilitate ALS research nationally. The National ALS Biorepository (Biorepository), a component of the Registry, collects samples across the United States via an in-home collection (e.g., blood, urine, or saliva) and postmortem collection (e.g., brain, bone, spinal cord, cerebrospinal fluid, muscle, and skin). This Biorepository is one of the largest in the country and collects pristine samples specifically for research; the few other existing ALS biorepositories largely have left-over samples from various clinics, medical practices, or previous clinical trials in the United States. Furthermore, the National ALS Biorepository specimens are collected from a geographically representative sample of Registry enrollees. The intent of the Biorepository is to collect specimens from at least one person per state. In addition, these de-identified samples can be paired with completed risk factor survey data (e.g., occupational and military history) from the Registry. Researchers are able to request samples alone or paired with risk factor data. The availability of additional specimens on a national sample of ALS patients further expands the research potential on the genetics, potential biomarkers, environmental pollutants, and etiology for ALS. Additional information for requesting samples and/or risk factor data is available at https://wwwn.cdc.gov/als/ALSRegistryResearchApplicationInfo.aspx.

The Registry also continues to fund ALS research nationally and internationally and across all disciplines of science to help determine etiology and risk factors. Since 2010, the Registry has funded 16 research projects, with three new R01 grants added in 2018. The goal behind this research portfolio is to better understand ALS in such areas as exposures to environmental pollutants, comparison of epigenetics in different population cohorts, exposure risks of cyanobacteria, and antecedent medical conditions (e.g., how chronic medical conditions and drugs might affect susceptibility to ALS). A complete list of funded studies is available at https://www.cdc.gov/als/ALSExternalResearchfundedbyRegistry.html#n. In addition, the Registry’s research notification system continues to inform and connect patients with clinical trials and epidemiologic studies. To date, approximately 40 institutions have used this system for patient recruitment. Information about this recruitment is available at https://www.cdc.gov/als/ALSResearchNotificationClinicalTrialsStudies.html.

The findings in this report are subject to at least three limitations. First, because ALS is not a nationally notifiable disease, the possibility of underascertainment exists and the three databases from which the majority of cases are identified are not representative of the U.S. population. The databases include a large percentage of persons aged ≥50 years; however, both the U.S. Department of Veterans Affairs and Medicare have special considerations that allow persons with ALS to receive benefits for ALS without waiting periods or meeting age requirements, increasing the likelihood that they are in the databases. In addition, the Registry seeks to use capture/recapture methodology for future reports to estimate the percentage of missing ALS cases, as well as to improve self-enrollment by increasing awareness and outreach in underrepresented populations identified in certain geographic areas (*8*,*9*). Second, although every attempt was made to deduplicate files, differences in fields collected from the various sources, misspellings of names, and data entry errors could have prevented records from merging correctly. However, it is unlikely that this occurred in numbers sufficient to affect the overall conclusions. Finally, the calculation of ALS incidence with Registry data is not possible at this time because the date of diagnosis is not captured through the large administrative database approach, and cases without a date of diagnosis account for 79.6% of cases in the Registry.

The establishment of the National ALS Registry and the National ALS Biorepository fills an important scientific gap by providing estimates of prevalence of this disease and facilitates further study of risk factors and etiology. Furthermore, the enhancements to the Registry also increase its potential for ALS research and detection of more cases. As more persons with ALS enroll and complete surveys, a better understanding of possible risk factors might emerge.

**Summary**

**What is already known about this topic?**

Amyotrophic lateral sclerosis (ALS) is a progressive and fatal neuromuscular disease with no cure.

**What is added by this report?**

In 2015, a total of 16,583 persons were identified as having definite ALS. The estimated prevalence of ALS in 2015 was 5.2 per 100,000 population, which is similar to that in 2014 (5.0). Case ascertainment was unaffected by the inclusion of *International Classification of Diseases, Tenth Revision* codes in the algorithm used to identify cases from national databases.

**What are the implications for public health practice?**

Through ongoing enhancements and expanded outreach and promotion, the National ALS Registry has the potential to facilitate ALS research and better describe the epidemiology of ALS cases in the United States.

## Footnotes

* ALS registry act of 2008, Pub. L. 110–373, 122 Stat 4047 (October 8, 2008). https://www.cdc.gov/als/files/ALS-Registry-Act-Public-Law-110-373.pdf.
